# The temporal basis of angiogenesis

**DOI:** 10.1098/rstb.2015.0522

**Published:** 2017-03-27

**Authors:** Katie Bentley, Shilpa Chakravartula

**Affiliations:** 1 Computational Biology Laboratory, Beth Israel Deaconess Medical Center, Harvard Medical School, Boston, MA, USA; 2 Cellular Adaptive Behaviour Laboratory, Rudbeck Laboratories, Department of Immunology, Genetics and Pathology, Uppsala University, Uppsala, Sweden

**Keywords:** time, dynamics, morphogenesis, adaptation, active perception, vascular

## Abstract

The process of new blood vessel growth (angiogenesis) is highly dynamic, involving complex coordination of multiple cell types. Though the process must carefully unfold over time to generate functional, well-adapted branching networks, we seldom hear about the *time-based* properties of angiogenesis, despite timing being central to other areas of biology. Here, we present a novel, time-based formulation of endothelial cell behaviour during angiogenesis and discuss a flurry of our recent, integrated *in silico/in vivo* studies, put in context to the wider literature, which demonstrate that tissue conditions can locally adapt the timing of collective cell behaviours/decisions to grow different vascular network architectures. A growing array of seemingly unrelated ‘temporal regulators’ have recently been uncovered, including tissue derived factors (e.g. semaphorins or the high levels of VEGF found in cancer) and cellular processes (e.g. asymmetric cell division or filopodia extension) that act to alter the speed of cellular decisions to migrate. We will argue that ‘temporal adaptation’ provides a novel account of organ/disease-specific vascular morphology and reveals ‘timing’ as a new target for therapeutics. We therefore propose and explain a conceptual shift towards a ‘temporal adaptation’ perspective in vascular biology, and indeed other areas of biology where timing remains elusive.

This article is part of the themed issue ‘Systems morphodynamics: understanding the development of tissue hardware’.

## 1. Introduction

**Figure 1.**
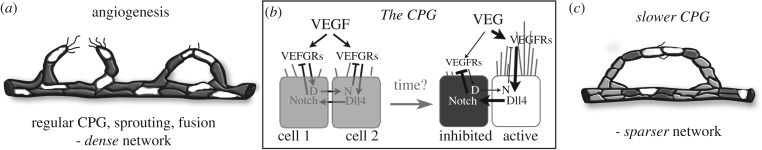
Angiogenesis and the ‘central pattern generator’ (CPG): key concepts. (*a*) New blood vessels sprout from pre-existing ones (active migratory cells, white; inhibited stalk cells, dark grey), tip cells leading sprouts fuse to form vessel loops that can support blood flow; reproduced with permission from [1]. This repeats to build the vascular network. (*b*) The CPG. VEGF/Notch signalling selects a heterogeneous pattern of active migratory cells and inhibited stalk cells from homogeneous (light grey) ECs. (*c*) Changing the speed that the CPG takes to select the active/inhibited (white/dark grey) phenotypes can alter vascular network structure as many more cells remain homogenous while they decide (light grey); adapted with permission from [1].

Temporal questions are integral to our everyday life: how long will it take? When is the deadline? Am I getting delayed by conversations in the hall? We are ever aware of our ability to be on time or late for events, and the role that local environment (e.g. obstacles or people talking in hallways) may play in affecting our punctuality. We also tend to appreciate that hard decisions take time to make; we set time aside for committees to discuss and deliberate before arriving at important decisions. However, in biology, the *time* it takes for cells to make ‘decisions’, or perform certain behaviours given their current local environmental conditions, is not always considered. This is particularly true in the field of vascular biology and in particular the study of angiogenesis (the specific process of blood vessel formation by ‘sprouting’ of new vessels from pre-existing ones) (figure 1*a*).

Angiogenesis is triggered in response to hypoxic tissue cues and is critical to the development of every tissue in the body, though each organ has markedly different vascular structures [2–4]. Pathological angiogenesis, where vessels can take on tortuous, bulbous and poorly branched morphologies are a hallmark of many diseases such as cancer and diabetic retinopathy [5–7]. Though angiogenesis is a highly dynamic and complex multicellular morphogenetic process, consensus on the biological mechanisms involved has been predominantly reached without consideration of the relative timing of events involved. In §2, we will describe the important initial process of cell competition during angiogenesis using a novel time-based formulation. In §3, we will discuss a number of studies arising from our collaborations with several independent experimental groups that show how temporal modulation of collective endothelial cell (EC) competition to migrate (specifically slowing it down, speeding it up and synchronizing it) can be achieved by modulating a surprising variety of molecular pathways and cellular processes/behaviours to generate different vessel shapes and network topologies. Together, these studies give a first glimpse towards a possible unification of disparate organ- and/or disease-specific vascular structures as *temporal adaptations* of a common pattern generating mechanism, which is simply temporally altered by the local tissue environment or prevailing conditions.

In several sub-disciplines of biological research, timing is very much of the essence; for example, research into the circadian rhythms that dominate our waking/sleeping cycles [8–10] or the Notch driven somite oscillatory clock that determines vertebrae spacing with every peak and trough [11–13] and intracellular spatio-temporal signalling such as calcium waves [14–16]. Historically, however, investigation in vascular biology can be considered to primarily have followed a ‘static paradigm’, largely due to the early necessity to rely on fixed tissue studies to observe vessels *in situ*. Recents advances in *in vivo* live imaging methods and fluorescent labelling tools for *in vitro* studies [17–19] have facilitated a shift towards the growing acquisition of dynamic datasets. Likewise, the increasing integration of computational simulations, where hypothesized mechanisms and processes are iterated over time, is providing a window into the nonlinear and often counter-intuitive nature of angiogenesis dynamics [20–26]. The dramatic difference in conceptual frameworks and perspectives taken by biologists specializing in different sub-domains or tissues can, in part, be understood as a result of the necessity to think within the practical bounds of ‘obtainable results’ by the prevailing tools used by that community. Even when tools and approaches advance, humans are, after all, creatures of habit, communities can take time to adapt, with studies driven by conceptual frameworks imposed by past limitations. Thus, we are careful here to make a distinction between (a) studies that obtain/utilize dynamic datasets, e.g. live imaging data or simulation results and (b) researching from a truly ‘*temporal* perspective’. This is because it is entirely feasible to formulate a study that yields dynamic datasets by using a ‘static perspective’; for example, if the central question of the study which utilizes dynamic imaging is ‘does gene X control behaviour Y?’ and not ‘what is the *relative timing* of different aspects of behaviour Y?’ or ‘*how long* does gene X take to trigger behaviour Y?’ Conversely, it is entirely feasible to answer temporal questions using static techniques. For example, if a spatial proxy for time delays in a system can be verified, static imaging is sufficient. This was masterfully demonstrated in [27], where a spatial distance was correlated to a specific gene expression time delay in the embryonic somite clock. Likewise, comparison between fixed tissues utilizing fluorescent-reporters with short versus long half-lives was recently used to demonstrate how Von Willebrand Factor (vWF) dynamically switches over long periods in the adult endothelium [28]. Thus to elucidate the temporal-basis of a process, we need to only start by asking time-based questions, and not necessarily be defined by our tools: *How long* does gene X take to *initiate* behaviour Y? *When* does behaviour Y occur, *before, after* or *during* behaviour Z? Can we *alter the rate* or *ordering* of behaviours Y and Z with gene X?

## 2. A novel time-based formulation of endothelial cell competition to migrate during angiogenesis

Endothelial cells, lining pre-existing blood vessels, migrate and proliferate to form new vessel ‘sprouts’ in response to hypoxia (low oxygen) signals from the surrounding tissue, such as vascular endothelial growth factor (VEGF). Sprouts must iteratively extend, lumenize and fuse, creating an immature plexus (figure 1*a*), then vessels either mature (e.g. by recruiting mural cells) or are pruned away (e.g. by reduced flow), the entire process requiring substantial cell rearrangement and heterogeneous collective migration, eventually establishing a functional, hierarchical vascular network perfusing the surrounding tissue with oxygen and nutrients [4]. In the initial stages of angiogenesis, a leading ‘tip cell’ must be selected from the pre-existing vessel, which will lead the growing sprout. Dll4-Notch lateral inhibition between neighbouring endothelial cells, in a feedback loop with VEGF–VEGFR (receptor) signalling is well characterized to be the ‘central pattern generating’ (CPG) mechanism by which migratory tip cells are selected (figure 1*b*) [3,29–32]. The tip cells inhibit their neighbouring cells, which are termed ‘stalk cells’. Stalk cells for a long time were considered to divide instead of migrating, following passively behind the tip to extend the sprout. However, the reality is far more complex and as the relative timing of cellular differences generated by the CPG change, so too can the network structure (figure 1*c*).

### (a) Time-ordering the VEGF/Notch ‘central pattern generator’

**Figure 2.**
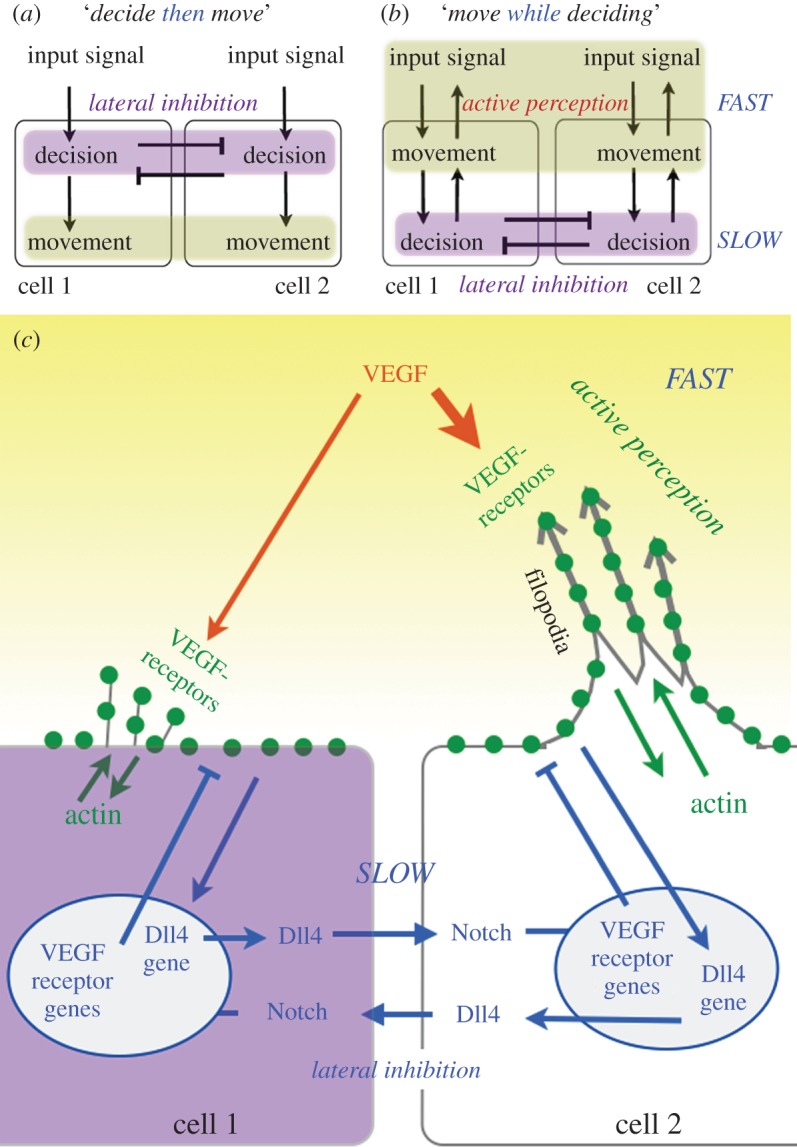
Time-ordering events and active perception. (*a*) Traditional feed-forward schematic view of the regulation of cell behaviour. (*b*) Schematic ordered chronologically reverses the flow. (*c*) The CPG spatio-temporally reveals active perception mechanisms: green dots indicate that sensors (VEGFR receptors) reside on the cell membrane, which is deformed and moved as actin polymerizes to form filopodia creating a sensorimotor loop (active perception).

In order to begin to understand how cell competition and the CPG unfold over time, it is worth first taking time to think about ‘the way we think about time’ in biology. An interesting cognitive barrier to formulating temporal questions about signalling processes could be argued to lie in the standardized nature of biological signalling diagrams. We will show that if we are careful to reorder such diagrams *chronologically* then ‘downstream and upstream’ can reverse, with important consequences for our understanding of how a biological process really works through time. ‘Signalling cascades’ are overarchingly presented, semantically and schematically, as ‘feed-forward’ processes, with the input signal located at the top of the diagram and the resulting output ‘behaviour’ located below. Even if the decision signalling network contains feedback loops, the overall process is represented as starting at the top and ending at the bottom. Time ordering of the events is subconsciously implied by the nature of how we culturally read information downwards on a page. To illustrate, consider the CPG in this style (figure 2*a*): a signal (VEGF) is detected by the EC in a pre-existing vessel by its VEGF-receptors (-1,-2 and -3); this triggers a ‘deciding process’ among the cells (Dll4-Notch lateral inhibition signalling between cells), as pVEGFR-2 (VEGFR-2 activation) upregulates Dll4. Notch1 is present on all ECs [33]. Dll4 then binds to Notch1 on the neighbouring cells, resulting in altered regulation of the VEGF-receptors and other factors via the Notch-target genes Hes1, Hey2 and Her1 [3], the net result being that the pVEGFR-2 level of the neighbouring cell is reduced, thus affecting the neighbouring cell's *ability to sense* VEGF. As the EC migratory responses (actin filopodia and lamella formation) are triggered by pVEGF-2 signalling, the end result is that the cell with the higher pVEGFR-2 and Dll4 will become migratory and the other cell is inhibited from movement due to Notch supressing VEGFR-2 activation. The implied temporal ordering of events in the classical schematic is: cells sense *then* decide *then* move.

#### (i) Stalk cell selection

Now let us consider the temporal reality (figure 2*b*). Lateral inhibition requires *multiple cycles* of gene regulatory changes to alter the synthesis of protein levels in order to amplify small differences between the cells' initial input signals [34]; each cycle has recently been shown to occur on the order of 4–6 h in ECs [35]. In contrast, the dramatic cell shape changes involved in EC migration, where filopodia and lamellapodia repeatedly extend and retract [36], occur much more rapidly (on the order of minutes) as they are powered by local activation and polymerization of sequestered actin at the cell surface, not gene expression in the nucleus [37–39]. It is thus clear that cell shape changes (filopodia) and early movements of the cell membrane must proceed *before* the establishment of a collective decision by lateral inhibition. Consequently, we propose that ‘move *while* deciding’ better captures the temporal ordering of the process, with early movement behaviours occurring *before* gene expression changes have taken place. This reversal of the schematic has several important implications; for example, filopodia are usually considered a feature of the *end* behaviour, and traditionally used to quantify fully selected tip cells [40], rather than a feature of the *initial* (‘deciding’) process occurring to some degree in all cells. Furthermore, how can the behaviour occur before it is selected? What about cause and effect? From a temporal perspective, it is easier to conceive that tip cells are not in fact ‘selected’ at all: all cells begin to migrate initially—it is their ‘default’ state [41]; it is the *stalk cells* that are selected by Notch signalling. With this realization, cause followed by effect is restored!

#### (ii) Active perception

As Dewey so elegantly stated in 1896 [42, pp. 137–138]: ‘*We begin not with a sensory stimulus, but with a sensorimotor co-ordination… In a certain sense it is the movement, which is primary, and the sensation which is secondary…’* We propose that filopodia and lamellapodia extension/retraction are a form of just such a sensorimotor coordination or ‘active perception’ by moving the cells' ‘sensors’ rapidly through the local environment they can better inform the cells on how to respond to the prevailing environmental conditions (figure 2*c*). In this way tissue-level feedback drives cellular decisions and adapts behaviour, rather than internal, genetic control dominating behaviour determination. Although critical for efficient complex decision-making and behaviour acquisition, such as language development in humans and visual guidance in robotics [43–46], active perception has received surprisingly little attention in cell biology as an aid to optimizing decision-making or cell behaviours, other than in adaptive systems research in relation to the flagellum of *E. coli* [47]. In a simulation study, we recently explored how active perception may be enhancing cell competition via the CPG and found that it provides a method to ‘temporally tune’, i.e. speed up, selection [48]. With filopodia, decisions to switch between tip and stalk cell migratory states exhibited ‘bistability’, which means switches are rapid and robust to small changes, whereas without filopodia they were slow and gradual, going through intermediate states. Thus, when cells ‘move *while* deciding’ decisions are kept fast and robust; if they were to ‘decide *then* move’ decisions would be extremely slow and the sprouts may not extend well during this time. The computational model used in this particular study has been utilized and extended across many different experimental collaborations described herein, providing critical insight into the complex dynamics of the CPG. In this model the EC outer membrane is represented at a subcellular level by a collection of individual computational agents (‘memAgents’) connected by springs following Hooke's law, which represents tension in the actin cortex beneath [49]. This ‘memAgent-Spring Model’ (MSM) allows simple, subcellular level rules to *generate* localized responses of individual memAgents on the cell surface by moving between on and off lattice modes, all together driving high resolution cell shape and gene expression changes in a realistic, *emergent* manner. Furthermore, the model was temporally well defined, in that all time delays involved were matched as far as possible to experimental data.

### (b) Time-ordering of cell rearrangement dynamics during sprouting

**Figure 3.**
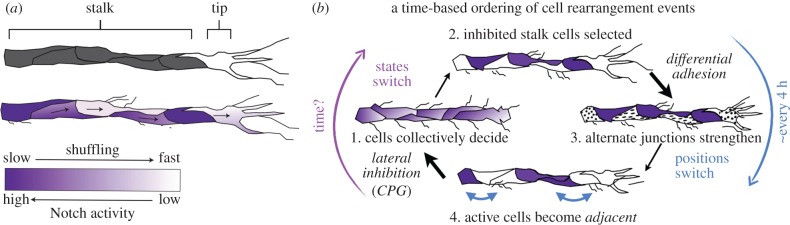
Cell rearrangement during sprouting. (*a*) (Top) The terms ‘tip’ and ‘stalk’ are positional, relating to regions of the sprout. (Middle and lower) Active migrating cells from within the stalk region rearrange positions at a rate inversely proportional to their Notch receptor activity. Reproduced with permission from [51]. (*b*) The interplay of the CPG and differential adhesion driven cell rearrangement making the timing explicit. This reveals that the length of time the CPG takes to re-establish differential states will be critical to determining the resulting network structure.

Experiments iteratively performed back and forth with the MSM model revealed that many of the (ECs) comprising the ‘stalk’ region of the sprout are far more ‘active’ and migratory than previously thought—where before only the tip cell was considered to be actively migrating [50]. Moreover, by considering the CPG chronologically as above, it becomes surprisingly intuitive that there will be other ‘active’ (migratory) cells interspersed along the vessel in between the selected stalk cells, and that they might try to move through it (figure 3*a*). In an *in vitro* embryoid-body sprouting assay, individual ECs were observed to switch positions within the stalk, overtaking the tip cell every 3.7 h on average. Similarly, Arima *et al.* [52] examined features of EC dynamics in an *in vitro* mouse aortic ring assay, terming it ‘cell-mixing’. Both studies experimentally demonstrated that Notch regulated this cell rearrangement behaviour.

To explore how Notch may regulate cell position changes and their timing, a substantial extension was made to the MSM model, incorporating cell–cell junction movements based on the widely used cellular Potts model (CPM) [53,54]. The principle of differential adhesion driven cell rearrangements is one that was particularly well demonstrated with the CPM in [54] and can be summarized as follows: differences in adhesion between two neighbouring cells confer a higher energy than if both cells were strongly adhesive [55]. Furthermore, two adjacent, weakly adhesive cells are considered to be in an even higher energy state. Random local junctional movements that minimize high-energy junctions and move the entire system to a lower energy state are more likely to be propagated. This principle was found to be sufficient to explain self-organization ‘cell-sorting’ phenomena of initially well-mixed populations of differentially adhesive cells into clusters where all strongly adhesive cells ‘stick together’, pushing out the weakly adhesive cells to the periphery [56]. VE-cadherin turnover was previously found to be higher in cells with higher pVEGFR-2 activation, which was rapidly reversible [57]. The MSM-CPM model predicted, and follow-up *in vitro* and *in vivo* experiments confirmed, that Notch regulates VE-cadherin adhesion between ECs such that *individual* junctions in the sprout have different adhesive properties [51]: active cells are weakly adhesive and inhibited stalk cells are more adhesive. This Notch regulated differential adhesion along the sprout was found to generate matching data to a wide range of experiments. In a later study using a different CPM-based model, Boas *et al.* [58] compared their data with those of Arima *et al.* [52] and discussed how cell mixing can be seen as an emergent property of the ‘sprouting system’ as a whole; thus both studies agreed that positional interchanges/jostling can spontaneously occur when cells have differential motion and adhesion, but Notch/VEGF signalling is required to regulate this in a way to match the precise dynamics seen *in vitro* by both experimental teams [58].

So what is the temporal ordering of events during angiogenesis when cells rearrange within the vessel and how is it so rigidly coordinated to permit normal networks to grow, rather than degenerating into a complex mess? From [41,51,59], we can formulate the following time-ordering (figure 3*b*): (i) cells are rapidly stimulated to extend migratory processes by VEGF; (ii) the CPG then selects inhibited stalk cells among the population exposed to VEGF, such that the remaining migratory cells are non-adjacently located and equally dispersed throughout the whole plexus (‘salt and pepper patterning’); (iii) this creates differential adhesion and junctional movements between neighbouring cells as the CPG regulates VE-cadherin and junctional stability; (iv) differential junctional activity drives cells to rearrange positions (this we know takes on average 3.7 h in total). Position switching must necessitate a disruption of the local alternating pattern of inhibited stalk cells, as differential adhesion self-organizes similarly adhesive cells to become adjacent (figure 2*a*). But as both active cells, high in Dll4, start to come into contact during their movement, the *ongoing* lateral inhibition in the CPG will have to act locally to re-establish a new salt and pepper pattern (step 1). Eventually, this will lead some cells to *switch* their migratory state to re-establish a salt and pepper pattern.

Thus with this time-ordering, the CPG coupled to the differential adhesion mechanism constitutes a collective level ‘active perception’ or ‘move *while* deciding’ mechanism where position-switching movements are part of the *precursor* for determining the selection of a new migratory state (based on the new local Dll4 environment encountered while moving through the other cells within the vessel). As an alternative theory, Beets *et al*. [60] have proposed a different time-ordering, following a more traditional ‘decide *then* move’ account of events. The Notch-target gene, Hes1, is known to autorepress itself in many other systems (though not all), which can lead to oscillations. Bmp-Smad signalling, which crosstalks with Notch-Dll4 signalling in ECs by also regulating Notch-target genes independently can itself lead to non-synchronous oscillations. Beets *et al.* [60] thus speculated that underlying non-synchronous oscillations which these pathways would cause in Notch-target gene regulation of VEGF-receptors and other factors among the ECs in the sprout, would provide an internal ‘molecular clock’ for cells to decide to regularly switch migratory states, leading to neighbours swapping positions, in time with the oscillations. Clearly, further study is needed to determine whether such a clock may exist, and whether it acts in competition or cooperation with any active perception processes.

### (c) How long do endothelial cells take to decide to rearrange positions within a growing vessel?

The next natural temporal question is how long does the CPG take to re-establish the salt and pepper pattern in order to drive the next round of positional interchanges (figure 3*b*)? In the ‘move while deciding’ mechanism above, the timing would be dependent on how long it takes the CPG to re-select the stalk cells to be fully inhibited and how different the cells' adhesion states need to be before junctional movements would begin to effectively move cells past each other again (i.e. How long can the CPG drive functional positional interchanges before it has fully finished the selection process?). Following the ‘decide then move’ time-ordering, rearrangements should presumably be quite regular, with the timing driven by gene expression delays in the Hes1 oscillator. In a related *in vivo* zebrafish study, Yokota *et al.* [61] asked the temporal question ‘*When* do ECs respond to VEGF?’ providing a tantalizing peak into exactly these kind of early selection dynamics. By utilizing the fast frame rates of lightsheet microscopy they live-imaged Ca^2+^ oscillations found to occur in VEGF–VEGFR-2 activated ECs during zebrafish intersegmental vessel (ISV) growth. They identified that ECs begin deciding early via the CPG while they reside within and across the entire dorsal aorta (DA), before a tip is selected to emerge as leader of a new sprout between somites. They also observed Ca^2+^ active cells being selected for the ‘stalk’ region of the sprout, which would follow up behind the tip cell in the sprout. As tip cells rarely overtake in the ISV sprout, they speculated that Notch signalling may be less effective once the cells are sprouting, leading to these adjacent active cells. However, cell rearrangement within the DA could be another explanation: the checkerboard of active cells initially selected in the DA by the CPG would drive positional interchanges along the DA, resulting in active cells becoming adjacent (figure 3*b*). If this occurred *concurrently* with the tip migrating up to form the sprout, one would observe the neighbouring stalk cell to be just as active. The amount of time it takes for the CPG to then re-establish a salt and pepper pattern within the somite space may be different, and take *longer* than it takes for the sprout to reach the top of the somite space and be complete. Indeed as we will discuss in §3, ISVs utilize an additional mechanism to create differential movement more quickly among neighbouring daughter cells after division, suggesting the CPG would be too slow alone in this region [62].

## 3. Changing the tempo to alter vascular structure

### (a) Slowing down selection alters vascular branching density

We first observed a curious phenomenon of the CPG ‘slowing down’ or more precisely ‘cells taking longer to collectively decide on their differential movement states' in a small simulation study on how Sirtuin 1 (SIRT1) loss drives a sparser branching phenotype in the mouse retina [7]. SIRT1 associates with the Notch intracellular domain (NICD), which becomes cleaved during Dll4 activation of Notch and travels to the nucleus to affect gene expression. SIRT1 functions as an NICD deacetylase, which opposes the acetylation-induced stabilization of NICD [7]. When SIRT1 is removed, NICD remains stable for longer, extending its effect on gene regulation by 2.5 times. Computational modelling, using the MSM model, integrated within this experimental study demonstrated that the CPG took longer to decide on the ECs' differential states because all cells were more inhibited from the start, meaning fewer filopodia extended from the cells. Simulations discussed earlier [48] indicated that anything that results in a reduction of the active perception ability of the cells will slow down the collective decision-making process as the cells will otherwise have more similar Dll4 levels without the additional positive feedback that active perception provides, which means more cycles through the CPG are required to amplify and generate the big differences needed to define differential states. However, once selected, tip cells were phenotypically normal, with many long filopodia because they were by then no longer receiving any further inhibition from their neighbours.

**Figure 4.**
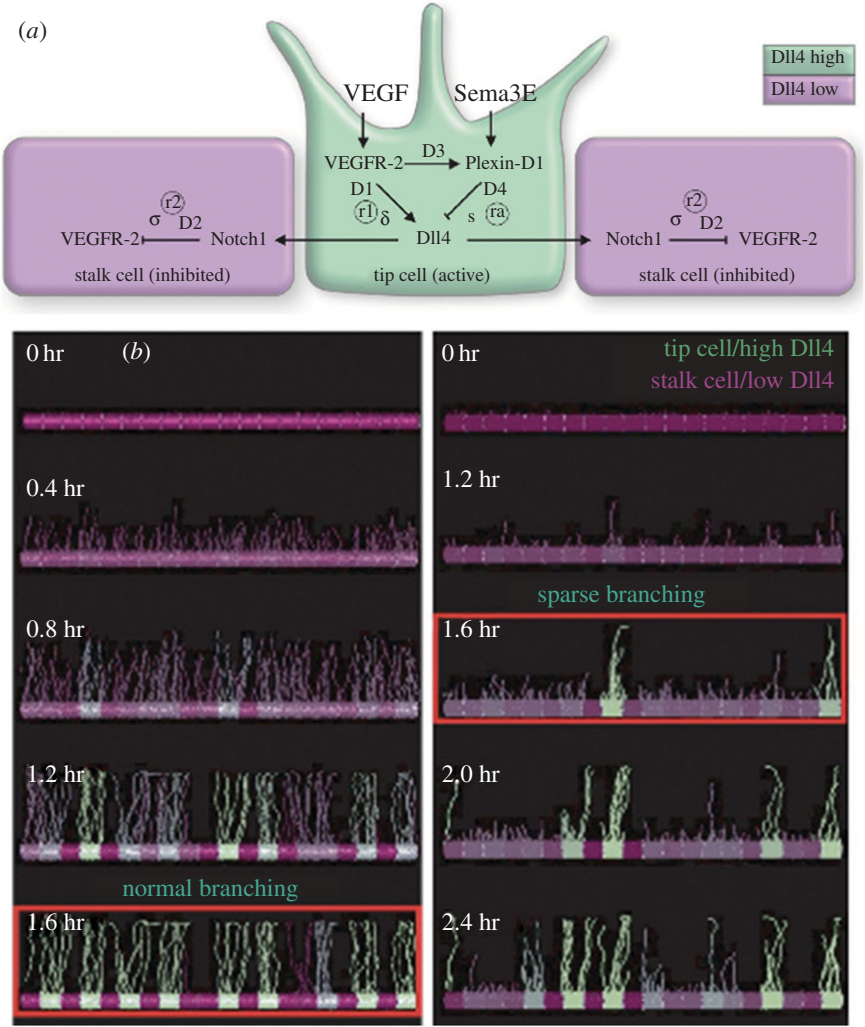
Sema3E-PlexinD1 speeds up the CPG increasing branching density (*a*) pathway schematic showing crosstalk with the CPG. (*b*) MSM simulations predict the sparser branching seen *in vivo* due to longer time spent collectively deciding states via increased lateral inhibition strength. Reproduced with permission from [1].

We wondered if this meant there could be a temporal explanation for other sparse branching phenotypes of vascular mutants and launched a full-scale study of the temporal dynamics involved in a pathway which produces a similarly sparse branching phenotype. Kim *et al.* [63] observed that Sema3E–Plexin-D1 signalling modulates VEGF-induced Dll4-Notch signalling via a feedback mechanism (figure 4*a*). Lack of Sema3E or Plexin-D1 resulted in an uneven vascular growth front, fewer tip cells, and a less dense network. Sema3E-Plexin-D1 interactions were simulated in the MSM model, which again predicted an overall delay in the CPG selection process [1]. Again, the elevated Dll4 levels, in the ECs drastically reduce the filopodia response in the first round of gene expression, forcing the system to rely solely on multiple cycles through lateral inhibition which, given the elevated Dll4 levels, also tends towards more oscillatory behaviour before a decision is reached (figure 4*b*; electronic supplementary material, figure S1*a*). The MSM-CPM model further predicted that ECs would take longer to switch positions during cell rearrangement in the sprout, by a factor of 1.26. To test these predictions experimentally, the Gu Laboratory developed a novel lung explant model for live imaging of individual cell dynamics during vessel sprouting within a *branched* network (as opposed to the linear sprouting assays used previously [50,52]). Consistent with the computational model predictions, live imaging quantification of the explant indeed showed a significant delay in the selection of new tip cells by a factor of 1.5 (defined as both initiation of a new sprout, and overtaking within an extending vessel) in Plxnd1−/− explants, with the tip cell selection frequency slowed down in the mutants (electronic supplementary material figure S1*b*,*c*). Finally, the topology of the live-imaged lung explant model was quantified and found to be significantly less branched. It is interesting to note that Sema3E-Plexin-D1 signalling as the intersection between somites and the DA was found to affect the collective dynamics of EC activation [61].

Thus, it is important to quantify how EC collective dynamics change as vessels extend through different closely located regions of tissue space, as timing may well change dramatically in different regions along the vessel if local conditions alter, driving local changes in vessel morphology and branching structure. We explored the concept of explicitly considering each cell’s ‘Umwelt’ or unique experience of its current local tissue environment in a recent ordinary differential equation (ODE) model of the CPG. Interestingly simulations predict that slowing down decisions in the CPG could sometimes counter-intuitively act to increase branching, if conditions are such that cells are more active during the long ‘indecisive’ period of time rather than being regularly over inhibited by elevated Dll4 levels as they were in the SIRT1 and PlexinD1 knock-out conditions. For example, a hypothetical SIRT1 gain-of-function simulation was performed, which leaves Notch signalling so weak, due to the rapid degradation of the NICD, that not only does it take a long time to select the stalk cells, but they are all highly migratory during this time [64].

### (b) Speeding up selection maintains sprout extension in proliferative tissues

We have seen that SIRT1 or Semaphorin loss can slow down selection in the CPG, reducing branching density (hyposprouting) *in vivo*. But how might cells speed up the CPG? Interestingly, the Herbert laboratory observed rapid assignment of differential motility in post-mitotic daughter cells in zebrafish ISVs [62]. Simulations of this scenario using the MSM model predicted that the CPG, even with normal levels of active perception (which is generated in this model by virtue of the simulated filopodia) would be too slow to account for the observed rapid selection. The Herbert laboratory further found this selection to be Notch-independent, as Dll4 morpholino fish also exhibited differential motility rates of daughter cells after cell division. Simulation of asymmetric division, where, most importantly, cell size and/or VEGF-receptor mRNA levels were unequally partitioned between daughters, was predicted to provide the most likely mechanism. Indeed, follow-up *in vivo* experiments confirmed that daughter cell size was unequal (due to the entire mitotic spindle consistently shifting to the proximal pole during metaphase) and VEGF-receptor mRNA levels were asymmetrically partitioned [62]. Thus, asymmetric division pre-biases the VEGF arm of the CPG so heavily that the slow selection process through Notch is no longer required, rapidly creating the differences needed to maintain the salt and pepper pattern of migratory cells in this fast moving system. This mechanism was found to be conserved in the mesencephalic veins of zebrafish, but only time will tell if it is required or utilized in other organs/organisms. Certainly, asymmetric division is a vital method for rapid differentiation of cell states in many other systems [65,66] including lymphatic progenitor mergence [67], which is also interestingly VEGF dependent.

### (c) Synchronized selection switches vessels from branching to expansion

**Figure 5.**
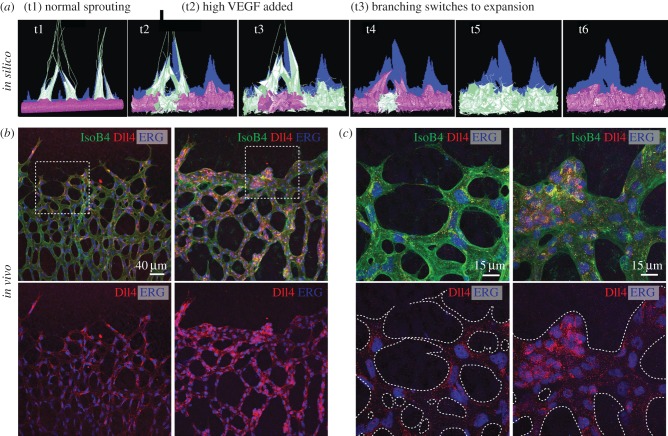
Pathologically high VEGF synchronized the CPG and promoted vessel expansion. (*a*) MSM simulation: t1 (time point 1) has a normal VEGF-A (linear gradient) extending above the sprout driving the CPG to rapidly select tip/stalk cells and promotes branching; high uniform VEGF-A levels are simulated from t2 through t6 mimicking VEGF-A intraocular injection in the mouse retina, switching the vessel to expand, not branch, with synchronizing oscillations in Dll4 (high Dll4, green; low Dll4, purple). (*b*) Images of the sprouting front of WT P5 mouse retinas not injected (wt) and injected with *mVegf165* (high VEGF injected) labelled with Dll4 protein (red) and endothelial cell nuclei (ERG; blue). (*c*) High magnification of (*b*). Reproduced with permission from [35].

Loss of temporal coordination in cell rearrangements is known to entirely disrupt embryogenesis and the extension of epithelial tubes [68–70], yet pathological disruption of cell rearrangements has only just begun to be explored in angiogenesis [35,51,71]. Early simulations with the MSM model predicted that the CPG would undergo a phase shift in its dynamics when VEGF levels become pathologically elevated, which is a hallmark of diseases such as cancer and retinopathies [5]. The model predicted that the negative feedback loop of the CPG becomes overloaded by the elevated input, maximally inhibiting the cells at each cycle through the pathway, removing the ability of the CPG to amplify small differences and instead ‘resetting all the cells back to full activation’ each cycle through. This had a marked effect on sprouting: now no clear sprouts emerged; instead adjacent cells attempted to migrate out, fused and then all switched to retract back in when inhibited [35,41,49] (figure 5*a*). This was a striking simulation prediction, and given the propensity of Notch signalling to oscillate in many other biological systems [12,72], it was put to the test. This prediction was recently experimentally confirmed across a plethora of experimental assays and disease conditions including EC monolayers and embryoid-body sprouting assays exposed to high VEGF-A (electronic supplementary material, figure S2), intraocular injection of VEGF-A in the mouse retina (figure 5*b*), the oxygen induced retinopathy (OIR) model of ischaemia driven ocular neovascularization, and finally syngenic mouse glioblastoma tumours [35]. Across all assays, Dll4 levels were observed to be either all high or all low within large contiguous clusters of ECs, and to fluctuate in synchrony between the two states with a periodicity of 4–6 h. In contrast, under normal VEGF conditions, Dll4 levels were found to asynchronously fluctuate among individual neighbouring cells as previously predicted by the MSM-CPM model and indeed hypothesized by Beets *et al.* [51,60].

Strikingly, this synchronization consistently correlated with a switch in vessel morphogenesis from branching to expansion in a proliferation independent manner. Intriguingly, cell-by-cell patterning of VE-cadherin at individual EC–EC junctions was also shown to switch from a differential pattern to large contiguous groups of cells with either all high or all low junctional activity in a number of different high VEGF tissues: glioblastoma vessels, mouse retinas intraocularly injected with high levels of VEGF-A or exposed to OIR [51]. Furthermore, simulations with the MSM-CPM model under high VEGF predicted a dramatic inability of the cells to rearrange/interchange positions due to the lack of difference between the motion/activity of individual cells [51]. Intercalation (positional interchanges during cell rearrangement) is known to drive lengthening of epithelial tubes and tissue in convergent extension [68,73], and we have found first evidence that without normal, differential cell rearrangements, when neighbouring cells become too similar/synchronized in their relative motion, position-switching is halted and blood vessels logically expand [35,51].

### (d) Targeting *time* for therapeutic normalization of vessels in disease

In almost every disease, of every organ, the vasculature plays a role. Inflammation responses and drug delivery depend on a good transport network, and in many diseases complications arise directly because the normal function or growth of the vasculature is perturbed [4,5]. Therapeutic treatments targeting abnormal morphogenesis of the vasculature, e.g. in cancer, have historically aimed to fully inhibit growth (anti-angiogenic therapy), primarily by targeting VEGF; however, many contraindications have now been identified [74]. *Normalization* therapy provides a promising alternative with good indications for a reduction in metastasis due to reduced hypoxia and improved drug delivery in the primary tumour [75], yet it remains unclear how to best achieve it. Notch signalling has shown promise as a cancer therapeutic and for normalization of arteriovenous malformations (AVMs) [76,77]; however, being a ubiquitous pathway, it likely cannot be directly targeted without incurring systemic side effects. Here, by taking a temporal perspective, we can see there are many alternative ways to alter Notch dynamics and vessel growth by targeting ‘temporal regulators’ of the CPG, opening up a new world of possible strategies to restore the balance, normalizing speed and the asynchronous nature of Notch dynamics required for normal tip cell competition, cell rearrangement, sprouting and vessel diameter. This approach was recently explored in an integrated study extending the MSM-CPM model to include metabolic regulation of cell movement. Cruys *et al*. [71] predicted, and experimentally confirmed, that a combination of VEGF inhibitors with a drug targeting glycolytic ATP production would normalize cell rearrangement in high VEGF pathologies, utilizing the mouse retinal angiogenesis model. EC metabolism is therefore proposed as a target combined with VEGF inhibitors to best normalize tumour vasculature by altering the temporal dynamics of differential adhesion and cell rearrangement, promoting branching rather than expansion.

## 4. Time to change our perspective?

**Figure 6.**
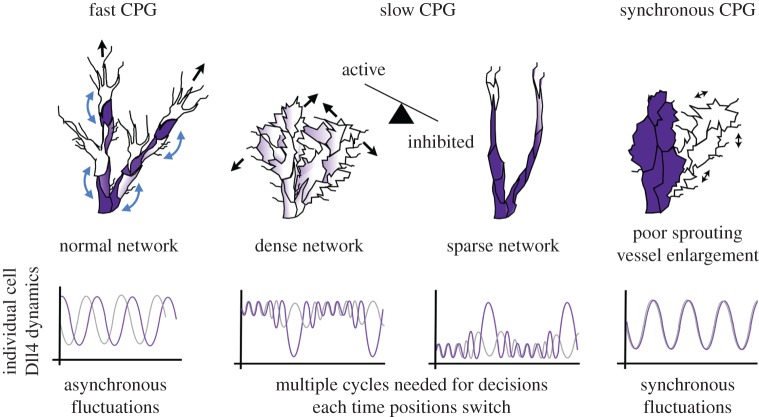
Temporal regulation of vascular patterning overview. Reproduced with permission from [35]. (Online version in colour.)

By specifically asking time-based questions and considering the real-time ordering of events involved in angiogenic sprouting processes, we are beginning to uncover a new world of ‘temporal regulators’ of cell competition whose modulation can dramatically alter sprouting morphogenesis and ultimately vascular tree structure and function (figure 6).

It is not always easy to shift our conceptual framework, or ask questions from a different perspective. The way we think, our unconscious assumptions and our choice of questions/approaches are always biased by our previous training and experience. This is why cross-disciplinary collaborations can be so important and illuminating, allowing us to see the problem from a different vantage point. If discipline training acts like a perceptual filter, reducing the potential solutions that we perceive exist to a given problem, to solve real-world complex problems we may ultimately need to build teams with members from many different disciplines. Greater integration of computer simulation and analysis methods in the future is ultimately critical to comprehending the complex and often counter-intuitive dynamics in biological systems as they unfold over time [78,79]. Computational simulations are essentially a means to specify events in a biological process in a sequential manner and, provided that temporal delays and assumptions are included, they can allow us to rigorously interrogate the dynamics in ways just not possible experimentally. Furthermore, the field of dynamic systems theory provides a wealth of tools and a mature knowledge base with which to rigorously explore and characterize the different dynamics possible in a biological system, as used in a small but growing number of studies of the endothelium [28,80]. Therefore, if even just a few more time-based questions are asked, and temporal quantifications made in experimental studies of the vasculature, our understanding of this dimension to the process will grow rapidly; integrated computer simulations can be built utilizing small numbers of temporal parameters to generate a plethora of new predictions and untangle complex dynamics ready for iterative experimental validation. Indeed, several temporal quantifications already made provide an opportune launch pad for new time-based studies in the future. For example, Arima *et al*. [52] found platelet-derived growth factor receptor antibody treatment, which reduces mural cell (pericyte) coverage of angiogenic sprouts, decreased mean tip duration and increased retrograde directional motility (away from the tip of the sprout), resulting in retarded branch elongation. Thus, the many other cells that interact with ECs may well alter the tempo in ways we need to understand next.

There are interesting parallels between the temporal modulation of angiogenesis by tissue environment factors (e.g. VEGF and Semaphorins) and recent revelations about the temporal regulation and ramifications of NF-κB oscillations [81]. NF-κB is a transcription factor (TF) that activates a variety of genes, some of which act to inactivate NF-κB, establishing oscillations. Recently, studies by Zambrano *et al.* [81] have shown that small perturbations in environmental conditions can gradually adjust the oscillations rates, causing the oscillations among neighbouring cells to synchronize. The authors propose that the need for NF-κB to shuttle back and forth in and out of the nucleus during these oscillations confers the cell with an incredible ability to wipe its *memory* (break regulation of genes biased by previous local tissue conditions and potentially adjust course in light of new conditions). They put forward a very interesting temporal hypothesis: ‘TF oscillatory dynamics is a means of segmenting time to provide renewing opportunity windows for decisions’, suggesting a temporal basis for adaptive behaviour. It is interesting to consider whether the CPG's temporal flexibility also confers ECs with an ability to perceive time past and present differently, permitting a very plastic and adaptive response in the complex and changing tissue environment during angiogenesis.

Although it has been noted that traditionally gene circuits were assumed to generate stable signals to constant external stimuli, the dynamics are now being revealed as more elaborate, including pulsed phases which again drive changes to behaviour [82]. The products of TF genes such as NF-κB also demonstrate a variety of temporal dynamics, accumulating quickly or slowly, oscillating themselves and all modulatable by external stimuli, with each class of gene related to specific behaviours unfolding sequentially or in specific time schedules [81]. In conclusion, it feels we are just still scratching the surface of how time is perceived, utilized and manipulated by cellular systems to generate complex adaptive behaviours. A wide, shimmering array of unexplored temporal regulatory factors and time-based cellular mechanisms likely remains to be discovered in angiogenesis and beyond.

## Supplementary Material

Electronic Supplementary Material
