# Supplementary material for: The temporal basis of angiogenesis
Source: Philos Trans R Soc Lond B Biol Sci. 2017 Mar 27;372(1720):20150522. doi: 10.1098/rstb.2015.0522 (PMC5379027; doi:10.1098/rstb.2015.0522)
Supplement: Electronic Supplementary Material [file rstb20150522supp1.docx]

**Electronic Supplementary Material**


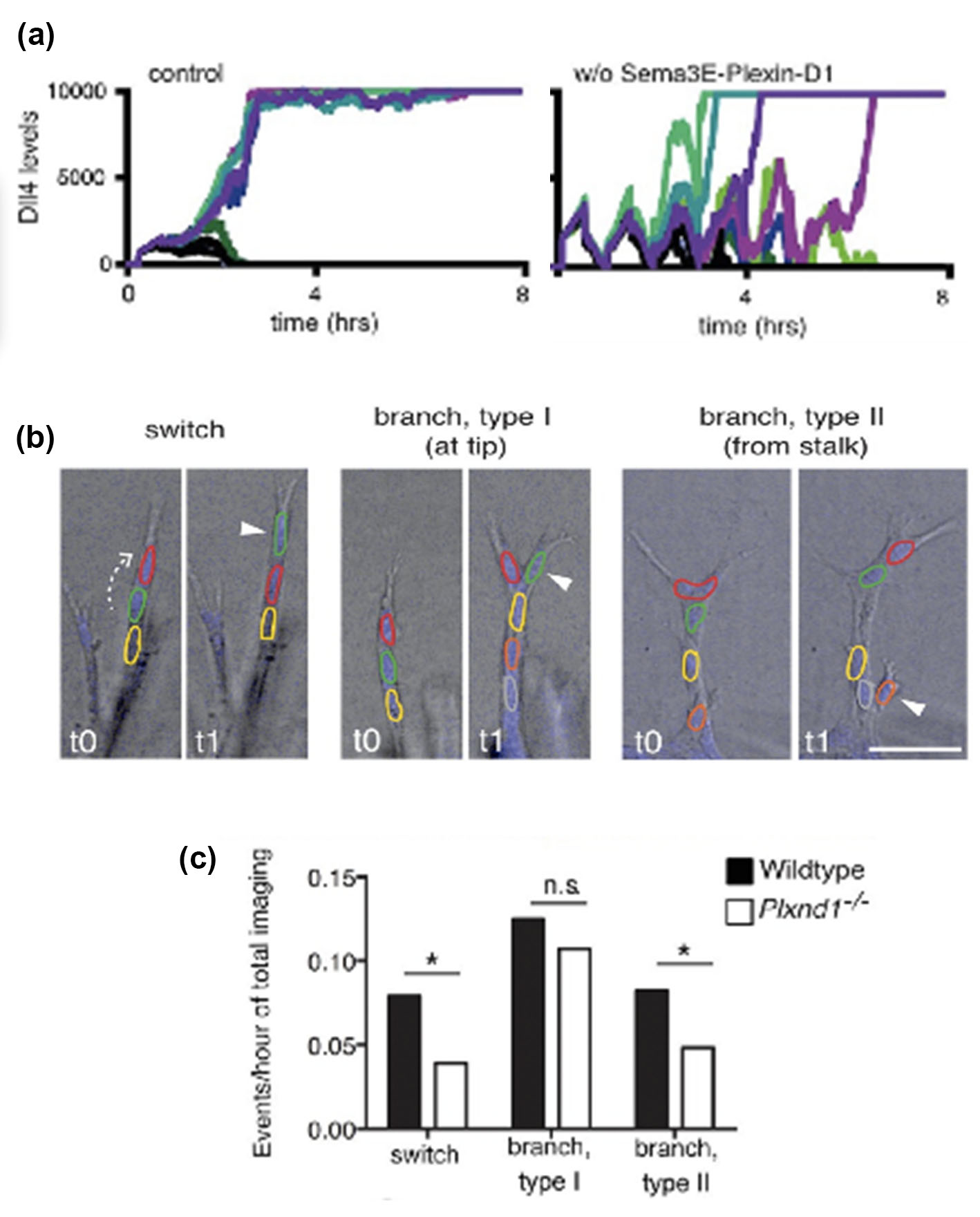


Figure S1. **Sema3E-PlexinD1 speeds up the CPG increasing branching density.** (a) MSM simulations predict sparser branching due to longer time spent battling in low level dll4 oscillations before amplifying differences enough to select tip/stalk states (b,c) Live imaging quantification of cell dynamics in sprouting mouse lung explants *ex vivo* show that tip cell selection (either by position interchanging, branching at the tip or from the stalk) frequency is lower: rearrangement and sprouting takes longer to occur in the absence of PlexinD1. All figures reproduced from [55] with permission.


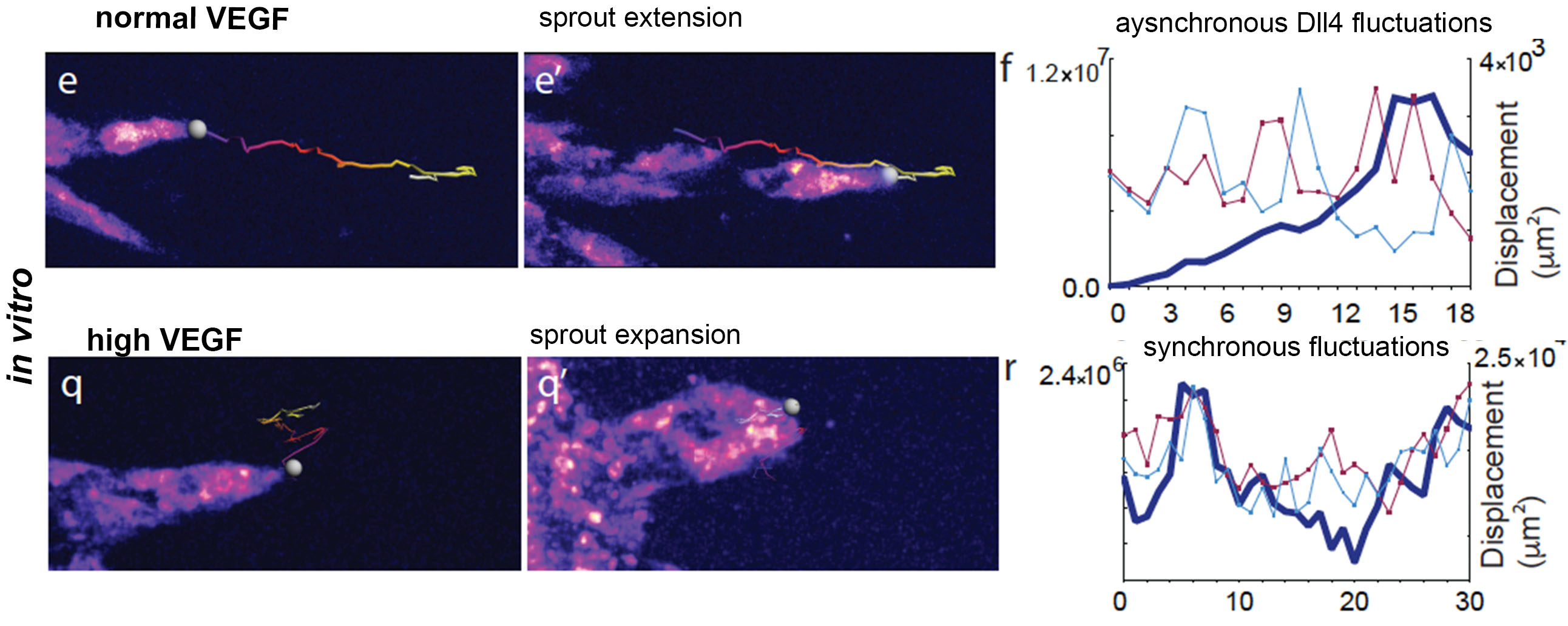


Figure S2. **Pathologically High VEGF synchronized the CPG and promotes vessel expansion**. (e,e’, q,q’) Start and end point of confocal time-lapse acquisitions of 3Dll4-dVenus (dynamic Dll4 reporter) embryoid bodies cultured in normal *Vegf* (50 ng/ml) and high *Vegf* (500 ng/ml). (f,r) Quantification by time-lapse microscopy of the sprout tip displacement in x,y,z dimensions (trailing multi-coloured lines on frames) y axis only – (thick blue line on graph) and Dll4 intensity at individual regions of the sprout.
